# Impact of COVID-19 on household hunger and socio-economic inequality in South Africa: a comparative analysis using NIDS-CRAM (2020–2021) and NFNSS 2022 data

**DOI:** 10.3389/fpubh.2025.1736131

**Published:** 2026-01-12

**Authors:** Akim Tafadzwa Lukwa, Plaxcedes Chiwire, Folahanmi Tomiwa Akinsolu, Paidamoyo Bodzo, Denis Okova, Sikelela Charles Maseko, Tholang Mokhele, Whadi-ah Parker, Vuyo Mjimba, Thokozani Simelani, Charles Hongoro

**Affiliations:** 1 Division of Family Medicine, Family, Community and Emergency Care (FaCE), Faculty of Health Sciences, University of Cape Town, Cape Town, South Africa; 2 Health Economics Unit, School of Public Health and Family Medicine, Faculty of Health Sciences, University of Cape Town, Cape Town, South Africa; 3 Western Cape Department of Health, Western Cape Province, Cape Town, South Africa; 4 Department of Health Services Research, CAPHRI Care and Public Health Research Institute, Maastricht University, Maastricht, Netherlands; 5 Department of Public Health, Faculty of Basic Medical and Health Sciences, Lead City University, Ibadan, Oyo, Nigeria; 6 Center for Reproduction and Population Health Studies, Nigerian Institute of Medical Research, Lagos, Nigeria; 7 Developmental, Capable and Ethical State, Human Sciences Research Council, Pretoria, South Africa; 8 School of Health Systems and Public Health (SHSPH), Faculty of Health Sciences, University of Pretoria, Pretoria, South Africa

**Keywords:** COVID-19 pandemic, Erreygers concentration index, food insecurity, household hunger, National Food and Nutrition Security Survey (NFNSS), NIDS-CRAM, socio-economic inequalities, South Africa

## Abstract

**Background:**

Food insecurity is a persistent socio-economic challenge in South Africa that was sharply exacerbated by the COVID-19 pandemic. This study compares household hunger during the acute pandemic period and the early recovery phase and examines how socio-economic inequalities in food security evolved.

**Methods:**

We analyzed five waves of the National Income Dynamics Study—Coronavirus Rapid Mobile Survey (NIDS-CRAM, 2020–2021) and the National Food and Nutrition Security Survey (NFNSS, 2022). A harmonized 7-day household hunger indicator was recoded as “no household hunger” and modeled using survey-weighted logistic regression. Socio-economic-related inequality in being hunger-free was assessed using the Erreygers Concentration Index and decomposition analysis, with sensitivity checks for alternative socio-economic status (SES) specifications and model diagnostics.

**Results:**

Hunger peaked at 26.47% in Wave 1 of NIDS-CRAM and declined to 16.07% by Wave 5, before falling to 8.19% in NFNSS. Improvements were uneven; several provinces, notably the Northern Cape, Free State and North West, remained comparatively food insecure. Across all waves and NFNSS, higher SES was strongly associated with a lower risk of hunger, and living in informal or traditional dwellings and larger household size were consistently associated with a higher risk of hunger. Erreygers indices were positive in all periods, indicating pro-rich inequality in food security that intensified during the pandemic and narrowed only modestly post-pandemic, with SES the dominant contributor.

**Conclusion:**

Although household hunger declined below pandemic peaks, the recovery in food security has been unequal and remains strongly patterned by socio-economic status and place, underscoring the need for structural, equity-focused policy responses.

## 1 Introduction

Food insecurity is one of the most pressing socio-economic challenges in South Africa, particularly in low-income and vulnerable communities (1–4). Defined as the lack of consistent access to adequate food due to economic or other resource constraints, food insecurity is both a cause and consequence of poverty (5). Over the years, South Africa has implemented several social protection programs, such as the Child Support Grant and the National School Nutrition Program, to alleviate hunger, especially among poor households (6). These programs have contributed to some progress in reducing household hunger, with the proportion of food-insecure households declining in the years leading up to the COVID-19 pandemic. However, deep-rooted inequalities in access to resources, employment, and basic services such as electricity and piped water have meant that food insecurity remains widespread, particularly among certain demographic groups and regions (7).

South Africa's history of inequality is closely tied to its legacy of apartheid, which has left many households, particularly those in rural areas, informal settlements, and townships, more vulnerable to economic shocks (7, 8). Structural barriers such as limited access to quality education, healthcare, and employment opportunities exacerbate the risk of food insecurity in these communities (9, 10). Pre-pandemic estimates by Statistics South Africa (Stats SA) indicated that about 11.6% of the population experienced regular hunger, with higher rates in provinces like the Eastern Cape and Limpopo, which have large rural populations (1). Addressing food insecurity, therefore, requires a multifaceted approach that goes beyond emergency food aid and tackles the underlying social and economic inequalities (11–13).

The outbreak of the COVID-19 pandemic in early 2020, which led to stringent lockdown measures to contain the virus, caused severe disruption to the South African economy (4). The national lockdown, one of the strictest globally, led to an abrupt halt in many sectors, particularly the informal economy, which provides livelihoods for a significant portion of the population (14). The restrictions disproportionately affected informal workers, small-scale traders, and those in precarious employment, as they lacked the savings or social protection needed to buffer against the sudden loss of income (8). Consequently, the lockdown exacerbated household food insecurity (15), with many households unable to afford basic food items due to diminished or lost incomes.

Data from the National Income Dynamics Study—Coronavirus Rapid Mobile Survey (NIDS-CRAM) highlighted the extent of the hunger crisis during the pandemic. According to NIDS-CRAM, household hunger surged during the early months of the pandemic, with 26.47% of households reporting that at least one member had gone hungry in the previous 7 days (15–20). This marked a significant increase compared to pre-pandemic levels, underscoring the immediate economic impact of the lockdown measures. The sharp rise among food insecurity was particularly pronounced in households headed by women, those with larger family sizes, and those with unemployed members (17). These groups, already vulnerable before the pandemic, faced even greater challenges as job losses, school closures, and supply chain disruptions strained their ability to secure sufficient food.

The government's response to the pandemic included emergency measures to mitigate the worst effects of the economic downturn. The Social Relief of Distress (SRD) grant, introduced as a temporary relief measure, provided a modest income to millions of unemployed individuals not covered by existing social protection programs. In addition, top-ups to the Child Support Grant were implemented to support vulnerable households with children (21). While these interventions were crucial in preventing further deterioration of food security, they were limited in scope and duration, particularly as the economic effects of the pandemic persisted beyond the initial lockdown (22). The gradual easing of lockdown measures and the reopening of the economy in late 2020 and 2021 did lead to a partial recovery in household incomes. Still, hunger levels remained significantly higher than pre-pandemic levels.

The National Food and Nutrition Security Survey (NFNSS), conducted in 2022, offers valuable insights into the longer-term effects of the pandemic on household hunger (23). Unlike the NIDS-CRAM data, which captured the immediate impacts of the pandemic, the NFNSS provides a post-pandemic snapshot of food security across South Africa. Preliminary findings from the NFNSS suggest that household hunger levels declined to 8.9% in 2022, indicating some degree of recovery as economic activity resumed and emergency social assistance programs took effect.

While existing studies using NIDS-CRAM data have effectively documented the acute rise in hunger and its association with factors like female-headed households, larger family size, and unemployment during the crisis (15–20), this study provides a critical extension. First, it moves beyond the acute pandemic period to analyse the post-pandemic recovery and persistence of food insecurity using the more recent Simelane et al. (54) data. Second, it employs a comparative longitudinal analysis, explicitly modelling and contrasting the socio-economic and demographic determinants of household hunger across the peak-pandemic (2020–2021) and post-pandemic (2022) periods. This approach allows us to assess not only changes in prevalence but also whether the drivers of hunger and the disproportionate burden on vulnerable groups transformed or persisted as the context shifted from emergency to recovery.

Given the complexity of these challenges, understanding the long-term impact of the COVID-19 pandemic on household food security requires an analysis that captures its evolving effects. This study aims to contribute to the growing body of research by conducting a multi-wave longitudinal and comparative analysis of household hunger in South Africa. Using nationally representative data, we trace the prevalence and socio-economic determinants of hunger across five waves of the NIDS-CRAM survey (2020–2021), which cover the acute and evolving phases of the pandemic, and compare this trajectory to the post-pandemic snapshot provided by the Simelane et al. (54). Specifically, this study seeks to: (1) assess the temporal dynamics of household hunger from the pandemic's onset into recovery, and (2) identify and compare the key socio-economic and demographic determinants of hunger across these multiple time points. By doing so, it provides evidence on the trajectory of recovery and the persistence or transformation of underlying vulnerabilities across different phases of the crisis, aiming to inform targeted policy interventions that address both immediate food insecurity and the structural inequalities that render specific households and regions disproportionately affected.

## 2 Methods

### 2.1 Data sources

This study drew on two nationally representative surveys from the South African National Income Dynamics Study–Coronavirus Rapid Mobile Survey (NIDS-CRAM), Waves 1–5 (2020–2021), and the National Food and Nutrition Security Survey (NFNSS) conducted in 2022.

#### 2.1.1 National Income Dynamics Study – Coronavirus Rapid Mobile Survey (nids-cram)

NIDS-CRAM is a rapid-response longitudinal telephone survey implemented during the COVID-19 pandemic and derived from the National Income Dynamics Study (NIDS^1^), a nationally representative household panel. Individuals interviewed in NIDS Wave 5 (24), formed the sampling frame. Because fieldwork had to comply with lockdown protocols, NIDS-CRAM interviewed only the original NIDS adults sample and did not enumerate all household members. Due to practical constraints, NIDS-CRAM (25–29) was designed as a rapid-response telephone survey, focusing on individual respondents rather than all household members (24). As face-to-face fieldwork was suspended during COVID-19, NIDS-CRAM did not attempt to re-enumerate entire households, instead interviewed the original NIDS respondent, a design choice explicitly made to preserve respondent safety and survey continuity. Sampling weights were updated for each wave to adjust for differential non-response and restore representativeness of the adult population.

Wave 1, conducted between May and June 2020, took place during stages 3 and 4 of the national lockdowns, while Wave 2, conducted between July and August 2020, was during the “advanced” stage 3 (24, 30, 31). Wave 3 was conducted during stages 2 and 1 between November and December 2020, followed by Waves 4 and 5 between February and March 2021 and April and May 2021, respectively, with the country in stage 1 by Wave 5 (24, 30, 31). The survey collected data on key variables such as household hunger, employment status, income, access to services (water and, electricity), and demographic information. The survey included 5 652, 4 476, 4 429, 4 208, and 4 341 individuals in Waves 1–5 (25–29), respectively, with a focus on hunger over the past 7 days.

#### 2.1.2 National Food and Nutrition Security Survey (NFNSS)

NFNSS is a nationally representative cross-sectional household survey implemented after the acute phase of COVID-19 (23). It employed a two-stage cluster sampling design:

- Stage 1: Selection of Small Area Layers (SALs) within districts using probability proportional to size (PPS). A total of 1,382 SALs were sampled across all provinces, with stratification by both administrative boundaries and Livelihood Zones (LHZs) to improve precision in food security estimates.
- Stage 2: Within each selected SAL, 30–35 households were sampled using simple random selection. Replacement households and clusters were used to maintain representativeness where needed.

Within each household, the household head and caregivers were interviewed. The final realised household sample was 34,575 households, with weights constructed to ensure national and provincial representativeness. The NFNSS integrates elements of the Household Economy Approach (HEA) and the food-security continuum, including HFIAS, the Household Hunger Scale (HHS), and the Coping Strategies Index. The NFNSS utilised the Southern African Vulnerability Assessment Committee (SAVAC)-endorsed methodology (2), integrating qualitative and quantitative research elements to assess food security and vulnerability. This approach enhances methodological and data triangulation, utilising the Household Economy Approach (HEA) and the food security continuum (32). The survey provides an understanding of food insecurity by capturing economic, social, environmental, and political components. It captures key indicators related to food access, socio-economic status, and access to basic services. Both surveys are nationally representative and include appropriate weighting for generalizability.

#### 2.1.3 Comparability and harmonisation of NIDS-CRAM and NFNSS

Although NIDS-CRAM and NFNSS differ in sampling frames (panel of adults vs. household cross-section) and data collection modes (telephone vs. face-to-face), both surveys are probability samples with design weights, allowing population-level inference. For this study, the primary comparability consideration is not sampling, but rather the measurement context of hunger:

- NIDS-CRAM uses a standalone 7-day household hunger item.
- NFNSS includes a 7-day household hunger item within the Coping Strategies Index module.

Both surveys, therefore, contain conceptually aligned 7-day hunger measures, allowing harmonisation into a single binary indicator of “any household hunger”. Nevertheless, because question framing and survey context differ, cross-dataset contrasts are interpreted descriptively rather than as strict before-and-after estimates of change.

### 2.2 Outcome variable: household hunger

The primary outcome for regression and inequality analysis was a binary indicator of no household hunger in the past 7 days. This was interpreted as a simple measure of household food security on the extensive margin (whether any hunger episode occurred), rather than a measure of severity or duration of hunger.

#### 2.2.1 NIDS-CRAM

In NIDS-CRAM Waves 1–5, household hunger was captured with the question, “*In the last 7 days, has anyone in your household gone hungry because there was not enough food?”* In the original datasets, the recoded variable household hunger is coded 0 = No and 1 = Yes (any household member went hungry). For this analysis, we constructed harmonised “no hunger” indicators for each wave as follows: *NoHunger* = 1 denotes no household hunger in the last 7 days, and 0 denotes any household hunger. These *NoHunger* variables were used as the dependent variables in the NIDS-CRAM logistic regressions and as the health outcome in the concentration index analysis.

#### 2.2.2 NFNSS

In NFNSS, household hunger over the past 7 days was measured with the question, “*In the past 7 days, have there been times when your household did not have enough food?”* In the recoded NFNSS variable, the coding is 0 = Yes (any episode of not having enough food) and 1 = No. To align the direction of the outcome with NIDS-CRAM, we generated a corresponding “no hunger” variable, *HH_nohunger_F* = *1 – HH_hunger_F* (for non-missing observations), so that *HH_nohunger_F* = *1* indicates no household hunger in the last 7 days and 0 indicates any household hunger. This variable was used as the dependent outcome in the NFNSS logistic regression models and as the bounded health variable in the Erreygers concentration index.

Using “no hunger” (rather than “any hunger”) as the outcome has two advantages. First, it aligns the interpretation of odds ratios across models: values above 1 indicate that factors are associated with better food security (i.e. higher odds of being hunger-free). Second, it allows a more straightforward interpretation of the Erreygers Concentration Index, where positive values indicate that the desirable outcome (no hunger) is disproportionately concentrated among richer households, and negative values indicate a pro-poor concentration of hunger. We note, however, that this binary outcome remains a simplification of the richer food security information available in NFNSS and does not capture hunger severity or duration; we therefore interpret results as pertaining to the presence or absence of any recent household hunger episode.

### 2.3 Explanatory variables

We selected predictors *a priori*, guided by established evidence on socio-economic vulnerability and food insecurity in South Africa. Variables were harmonised across datasets where needed.

- Sex of household head (male/female)
- Age of household head (continuous)
- Household size (continuous)
- Employment status (economically inactive, unemployed, employed)
- Race (Black African, Coloured, Asian/Indian, White)
- Education (primary, secondary, tertiary)
- Socio-economic status (SES): survey-specific wealth/income quintiles (1 = poorest; 5 = richest)
- Dwelling type (formal; traditional/mud; informal shack)
- Access to basic services: electricity (yes/no), piped water (yes/no)

These variables are summarised in Table 1.

**Table 1:** Independent variables.

**Variable**	**Categories**
Sex of household head	Male or Female
Age of household head	Continuous variable
Household size	Continuous variable
Employment status of household head	Economically inactive, unemployed, or employed
Race of household head	Black African, people of color, Asian/Indian or White
Highest education level of household head	Primary, Secondary or Tertiary
Household socio-economic status (SES)	Quintiles 1 (poorest), 2 (poorer), 3 (middle), 4 (richer) and 5 (richest)
Dwelling type	Formal, traditional or informal housing
Access to electricity	Yes or No
Access to piped water	Yes or No

### 2.4 Statistical analysis

All analyses were conducted using Stata 17, and survey weights were applied to adjust for the complex sampling design of both NIDS-CRAM and NFNSS datasets.

#### 2.4.1 Descriptive statistics

We computed weighted estimates of household hunger prevalence by wave (NIDS-CRAM), province, SES quintile, and other characteristics. We also compared NIDS-CRAM (pandemic period) with NFNSS (post-pandemic period).

#### 2.4.2 Logistic regression analysis

To identify socio-economic determinants of hunger, we fitted theory-driven multivariable logistic regression models separately for:

- Each NIDS-CRAM wave (1–5), and
- NFNSS.

Model covariates were selected *a priori*, following conceptual frameworks on poverty, household vulnerability and food access (33, 34). All models were estimated with survey probability weights. We report adjusted odds ratios (aORs) with 95% confidence intervals. A sensitivity analysis modelled SES as an ordinal variable (poorest to richest).

#### 2.4.3 The Erreygers concentration index and decomposition analysis

The Erreygers Concentration Index measures inequality in a health variable (household hunger) linked to socio-economic status and takes values between −1 and +1 (35). In this study, the health variable is “*no household hunger in the past 7 days”*, a desirable outcome indicating that the household did not experience any hunger episode. An ECI value of 0 denotes the absence of socioeconomic-related inequality. A positive ECI indicates that the desirable outcome (being hunger-free) is disproportionately concentrated among richer households (a pro-rich distribution of food security). Conversely, a negative ECI indicates that being hunger-free is more concentrated among poorer households (a pro-poor distribution of food security).

The Erreygers Index was calculated for each NIDS-CRAM wave (Waves 1–5) and for the post-pandemic period captured in the Simelane et al. (54). To understand the drivers of these inequalities, each ECI was decomposed into contributions from the socio-economic and demographic factors included in the regression models. This decomposition quantifies the extent to which each factor explains the observed inequality in the distribution of the “no hunger” outcome. A positive contribution indicates that the factor increases pro-rich inequality in food security. In contrast, a negative contribution indicates that the factor reduces inequality or contributes to a pro-poor distribution.

The decomposition follows two steps:

- Regression stage: the health outcome is modelled as a function of explanatory variables (e.g., SES, household size, education).
- Decomposition stage: each factor's contribution is computed as the product of its marginal effect, its mean, and its own concentration index, expressed as a share of the overall ECI.

##### Step 1

A regression model links the health outcome to explanatory factors (such as socio-economic status, age, education, etc.). The basic form of the regression equation is:


$$\begin{array}{l} y_{i}\;=\;a+\underset{k}{\sum }\beta_{k}\;X_{ki}+\varepsilon_{i} \end{array}$$

where;

*y*_*i*_ is the health outcome for household *i*

*X*_*ki*_ represents the *k-th* explanatory factor (e.g., SES, education level) for household *i*

β_*k*_is the is the coefficient (impact) of the *k-th* factor on the health outcome

ε_*i*_ is the error term for household *i*

##### Step 2

The contribution of each factor to the overall concentration index is calculated using the product of its regression coefficient and the concentration index of that factor.


$$\begin{array}{l} CI\;=\;\underset{k}{\sum }\left(\frac{\beta_{k}\;x_{k}}{y}\right){CI}_{k}+\frac{{GCI}_{\varepsilon }}{y} \end{array}$$

where;

*x*_*k*_ is the mean of the *k-th* factor

y is the mean of the health outcome

CI_k_ is the concentration index of the *k-th* explanatory factor

GC_ε_ is the generalised concentration index of the error term ϵ, representing the unexplained inequality.

## 3 Results

### 3.1 Descriptive statistics

Table 2 presents the proportions of households reporting no household hunger across the NIDS-CRAM Waves 1–5 and the NFNSS. The results show a clear improvement in food security over time. In Wave 1, 77.69% of households reported no hunger in the previous 7 days (95% CI: 76.17–79.14). This proportion increased steadily to 84.01% in Wave 2 (95% CI: 82.53–85.38), then declined slightly to 81.59% in Wave 3 (95% CI: 80.08–83.02). Waves 4 and 5 again showed improvements, with 83.30% (95% CI: 81.69–84.80) and 83.93% (95% CI: 82.43–85.33) of households, respectively, reporting no hunger. In comparison, the NFNSS showed a substantially higher proportion of households with no hunger 91.81% (95% CI: 91.47–92.14). This suggests that, post-pandemic, food security had improved beyond levels observed during the early phases of COVID-19.

**Table 2:** Percentage of households with no household hunger in NIDS-CRAM Waves 1–5 and the NFNSS.

**Variable**	**Observations (*n*)**	**Proportion (%)**	**Std. Err**.	**[95% Conf. Interval]**
Wave 1	7 012	77.69	0.0076	[76.17, 79.14]
Wave 2	5 636	84.01	0.0073	[82.53, 85.38]
Wave 3	6 106	81.59	0.0075	[80.08, 83.02]
Wave 4	5 611	83.30	0.0079	[81.69, 84.80]
Wave 5	5 853	83.93	0.0074	[82.43, 85.33]
NFNSS	30 891	91.81	0.0017	[91.47, 92.14]

The “no household hunger” indicator is coded 1 = no household member went hungry in the past 7 days; 0 = any household hunger episode. Figures are weighted using survey design weights for each dataset.

#### 3.1.1 Provincial differences in no-hunger prevalence

Table 3 summarises the percentage of households that did not experience hunger across provinces in all NIDS-CRAM waves and in the NFNSS. Provincial patterns reveal consistent improvements over time within NIDS-CRAM, but also substantial variation across regions. In the Western Cape, the proportion of households with no hunger increased from 81.46% in Wave 1 (95% CI: 76.01–85.91) to 88.56% in Wave 5 (95% CI: 83.12–92.41). NFNSS levels were even higher (99.25%; 95% CI: 98.89–99.49), reflecting strong post-pandemic recovery. The Eastern Cape also showed improved food security, rising from 76.01% in Wave 1 (95% CI: 71.18–80.25) to 84.91% in Wave 5 (95% CI: 80.39–88.53). NFNSS again indicated very high levels of food security (99.27%; 95% CI: 99.02–99.46).

**Table 3:** Proportions of households NOT experiencing hunger (No Hunger) by region across the NIDS-CRAM 1–5 waves and NFNSS, reported as percentages with standard errors and 95% confidence intervals.

**Region**	**Wave 1**			**Wave 2**			**Wave 3**			**Wave 4**			**Wave 5**			**NFNSS**		
	**Prop**.	**SE**	**[95% CI]**	**Prop**.	**SE**	**[95% CI]**	**Prop**.	**SE**	**[95% CI]**	**Prop**.	**SE**	**[95% CI]**	**Prop**.	**SE**	**[95% CI]**	**Prop**.	**SE**	**[95% CI]**
Western Cape	81.46%	0.0252	[76.01, 85.91]	87.34%	0.0233	[82.04, 91.24]	87.93%	0.0234	[82.54, 91.81]	89.00%	0.0262	[82.72, 93.19]	88.56%	0.0234	[83.12, 92.41]	99.25%	0.0015	[98.89, 99.49]
Eastern Cape	76.01%	0.0232	[71.18, 80.25]	83.51%	0.0207	[79.04, 87.18]	77.00%	0.0254	[71.66, 81.60]	82.27%	0.0218	[77.59, 86.15]	84.91%	0.0207	[80.39, 88.53]	99.27%	0.0011	[99.02, 99.46]
Northern Cape	71.93%	0.0322	[65.20, 77.80]	86.83%	0.0253	[81.02, 91.06]	79.05%	0.0323	[72.01, 84.69]	82.96%	0.0279	[76.78, 87.75]	88.42%	0.0205	[83.74, 91.88]	68.78%	0.0094	[66.92, 70.59]
Free State	72.98%	0.0280	[67.16, 78.10]	84.44%	0.0257	[78.71, 88.84]	84.76%	0.0212	[80.14, 88.46]	82.15%	0.0228	[77.24, 86.19]	81.68%	0.0248	[76.31, 86.05]	71.81%	0.0094	[69.93, 73.60]
KwaZulu-Natal	72.79%	0.0154	[69.68, 75.70]	77.79%	0.0155	[74.61, 80.67]	73.12%	0.0157	[69.93, 76.09]	78.46%	0.0155	[75.27, 81.34]	78.35%	0.0154	[75.19, 81.21]	99.80%	0.0004	[99.70, 99.87]
North West	67.60%	0.0340	[60.62, 73.88]	77.39%	0.0329	[70.30, 83.19]	78.77%	0.0261	[73.21, 83.43]	82.42%	0.0254	[76.89, 86.85]	79.36%	0.0275	[73.44, 84.24]	61.39%	0.0127	[58.87, 63.85]
Gauteng	82.55%	0.0164	[79.09, 85.54]	85.35%	0.0171	[81.67, 88.40]	85.86%	0.0165	[82.30, 88.80]	84.49%	0.0197	[80.22, 87.97]	85.51%	0.0180	[81.63, 88.69]	99.17%	0.0017	[98.77, 99.44]
Mpumalanga	77.90%	0.0214	[73.43, 81.80]	85.42%	0.0196	[81.14, 88.86]	77.94%	0.0249	[72.68, 82.43]	78.67%	0.0254	[73.26, 83.23]	81.18%	0.0224	[76.39, 85.18]	–	–	–
Limpopo	80.59%	0.0218	[75.97, 84.51]	89.64%	0.0151	[86.29, 92.25]	86.67%	0.0185	[82.62, 89.90]	87.77%	0.0195	[83.41, 91.10]	87.81%	0.0167	[84.14, 90.72]	–	–	–

Proportions are reported as percentages | Confidence Intervals (CI) are reported in square brackets | standard errors (SE).

In the Northern Cape, the pattern differed. Although no-hunger levels improved during NIDS-CRAM from 71.93% in Wave 1 (95% CI: 65.20–77.80) to 88.42% in Wave 5 (95% CI: 83.74–91.88), the NFNSS reported only 68.78% (95% CI: 66.92–70.59) of households with no hunger. The Free State showed moderate improvements within NIDS-CRAM, rising from 72.98% in Wave 1 (95% CI: 67.16–78.10) to 81.68% in Wave 5 (95% CI: 76.31–86.05). The NFNSS estimate was substantially lower (71.81%; 95% CI: 69.93–73.60), indicating a possible post-pandemic decline in food security.

In KwaZulu-Natal, improvements were modest, with no-hunger prevalence increasing from 72.79% in Wave 1 (95% CI: 69.68–75.70) to 78.35% in Wave 5 (95% CI: 75.19–81.21). NFNSS estimates were nearly universal (99.80%; 95% CI: 99.70–99.87). The North West province maintained comparatively low no-hunger levels throughout NIDS-CRAM, increasing from 67.60% in Wave 1 (95% CI: 60.62–73.88) to 79.36% in Wave 5 (95% CI: 73.44–84.24). However, NFNSS indicated only 61.39% (95% CI: 58.87–63.85) with no hunger, marking this province as one of the most food-insecure post-pandemic. In Gauteng, no-hunger prevalence increased from 82.55% in Wave 1 (95% CI: 79.09–85.54) to 85.51% in Wave 5 (95% CI: 81.63–88.69), with NFNSS reflecting near-universal food security (99.17%; 95% CI: 98.77–99.44). In Mpumalanga, no-hunger levels showed fluctuations but improved slightly from 77.90% in Wave 1 (95% CI: 73.43–81.80) to 81.18% in Wave 5 (95% CI: 76.39–85.18). NFNSS did not include comparable provincial estimates. Similarly, Limpopo experienced increases from 80.59% in Wave 1 (95% CI: 75.97–84.51) to 87.81% in Wave 5 (95% CI: 84.14–90.72), with no NFNSS estimate available.

Overall, most provinces showed rising proportions of households without hunger over the NIDS-CRAM waves, suggesting a gradual recovery during the pandemic period. The NFNSS generally indicated higher no-hunger levels in most provinces except the Northern Cape, Free State, and North West, where food security appeared to be deteriorating or remaining comparatively low.

### 3.2 Regression

Table 4 presents the results of the logistic regression models examining factors associated with households not experiencing hunger (No Hunger) across NIDS-CRAM Waves 1–5 and the NFNSS. Employment status showed some association with household food security, although the pattern was not entirely consistent over time. Compared with households where the respondent was not economically active, unemployed respondents generally had lower odds of not experiencing hunger in the early waves, with ORs of 0.66 (95% CI: 0.48–0.91) in Wave 1 and 0.65 (95% CI: 0.43–1.00) in Wave 2. This suggests greater vulnerability to hunger among unemployed households during the initial stages of the pandemic. By Waves 4 and 5, these associations attenuated, with ORs closer to or above 1 (e.g., 1.11; 95% CI: 0.75–1.63 in Wave 4; 1.38; 95% CI: 0.56–3.44 in Wave 5), although confidence intervals were wide and included 1. Employed respondents did not differ significantly from those not economically active in most waves, and NFNSS estimates also showed no clear association between employment status and food security, reflecting considerable uncertainty in post-pandemic patterns.

**Table 4:** Logistic regression for households NOT experiencing hunger (No Hunger) for NIDS-CRAM Waves 1–5 and NFNSS survey.

**Variable**	**Wave 1**	**Wave 2**	**Wave 3**	**Wave 4**	**Wave 5**	**NFNSS**
**Employment status (Ref: not economically active)**						
Unemployed	0.66 [0.48 – 0.91]^b^	0.65 [0.43–1.00]^c^		0.74 [0.51–1.07]	1.11 [0.75–1.63]	1.38 [0.56–3.44]
Employed	0.85 [0.62–1.16]	0.87 [0.58–1.31]	1.00 (omitted)	0.92 [0.62–1.36]	1.23 [0.83–1.83]	0.72 [0.27–1.92]
**Race (Ref: African/Black)**						
People of Color	1.34 [0.84–2.16]	2.25 [0.93–5.47]	4.01 [1.69–9.55]^a^	1.43 [0.70–2.93]	1.70 [0.74–3.92]	0.46 [0.06–3.40]
Asian/Indian	2.12 [0.69–6.50]	1.21 [0.36–4.10]		2.11 [0.46–9.68]	7.42 [1.51–36.51]^b^	
White	3.67 [1.17–11.53]^b^	7.26 [2.07–25.39]^a^	3.33 [0.47–23.53]	2.04 [0.71–5.87]	5.99 [1.96–18.38]^a^	0.84 [0.10–6.80]
**Socio-economic status (Ref: Poorest)**						
Poorer	1.06 [0.77–1.47]	1.58 [1.09–2.31]^b^	1.07 [0.58–2.00]	2.69 [1.89–3.83]^a^	1.55 [1.07–2.23]^b^	2.98 [1.18–7.53]^b^
Middle	1.62 [1.14–2.29]^a^	2.00 [1.31–3.05]^a^	1.29 [0.68–2.44]	2.19 [1.41–3.41]^a^	2.02 [1.37–2.99]^a^	3.46 [1.22–9.80]^b^
Richer	1.51 [1.07–2.13]^b^	2.67 [1.73–4.11]^a^	1.86 [0.88–3.90]	3.05 [2.02–4.62]^a^	4.96 [3.22–7.64]^a^	2.90 [1.13–7.42]^b^
Richest	3.75 [2.43–5.79]^a^	9.45 [5.53–16.12]^a^	5.29 [1.91–14.60]^a^	11.93 [6.62–21.48]^a^	10.06 [5.70–17.74]^a^	6.80 [2.23–20.72]^a^
**Dwelling type (Ref: House/Flat)**						
Tradition/mud	0.91 [0.64–1.28]	0.87 [0.62–1.23]	0.56 [0.30–1.07]^c^	0.89 [0.62–1.28]	0.64 [0.44–0.93]^b^	
Informal/shack	0.71 [0.50–1.00]^b^	0.69 [0.43–1.11]	0.57 [0.27–1.18]	0.69 [0.46–1.04]^c^	0.44 [0.28–0.69]^a^	1.38 [0.57–3.31]
**Electricity access (Ref: No)**						
Yes	1.33 [0.84–2.12]	0.75 [0.44–1.25]	1.94 [0.75–5.05]	1.08 [0.62–1.87]	1.58 [0.89–2.81]	1.11 [0.45–2.73]
**Piped water access (Ref: No)**						
Yes	0.89 [0.67–1.17]	0.94 [0.69–1.29]	0.45 [0.26–0.79]^a^	0.91 [0.69–1.21]	1.04 [0.79–1.37]	1.58 [0.76–3.28]
**Education (Ref: no education)**						
Primary education	1.41 [0.79–2.53]	1.10 [0.58–2.08]	0.49 [0.11–2.11]	2.01 [0.96–4.20]^c^	0.74 [0.39–1.41]	2.84 [0.35–23.19]
Secondary education	1.94 [1.05–3.57]^b^	1.24 [0.62–2.49]	0.71 [0.16–3.16]	1.89 [0.89–4.02]^c^	0.65 [0.32–1.31]	3.73 [0.41–33.64]
Tertiary education	6.62 [1.40–31.33]^b^	11.46 [2.19–59.91]^a^	0.45 [0.06–3.27]	5.46 [1.24–24.01]^b^	0.90 [0.20–3.95]	2.56 [0.22–29.30]
**Sex (Ref: Male)**						
Female	1.03 [0.81–1.32]	1.04 [0.77–1.40]	1.01 [0.62–1.65]	1.00 [0.74–1.34]	1.08 [0.81–1.43]	1.93 [0.96–3.88]^c^
Household size	0.97 [0.94–1.01]	0.98 [0.94–1.02]	0.97 [0.89–1.06]	0.89 [0.85–0.94]^a^	0.91 [0.87–0.95]^a^	1.15 [1.02–1.30]^b^
Respondent age	1.01 [1.00–1.02]	0.99 [0.98–1.00]^b^	0.99 [0.97–1.01]	0.99 [0.98–1.00]	1.00 [0.99–1.02]	0.98 [0.96–1.01]

Confidence intervals in parentheses

^a^*p* < 0.01, ^b^*p* < 0.05, ^c^*p* < 0.1.

Blank cells indicate variables which were omitted from the model due to perfect prediction or collinearity.

An OR > 1 indicates higher odds of NOT experiencing hunger, while OR < 1 indicates lower odds of NOT experiencing hunger.

Racial inequalities in food security were evident, particularly in the NIDS-CRAM waves. Using African/Black households as the reference group, People of Colour, Asian/Indian, and White households generally had higher odds of not experiencing hunger, although estimates were sometimes imprecise. For example, White households had substantially higher odds of not experiencing hunger in Wave 1 (OR: 3.67; 95% CI: 1.17–11.53) and Wave 2 (OR: 7.26; 95% CI: 2.07–25.39), with elevated odds also observed in Waves 3–5, including 5.99 (95% CI: 1.96–18.38) in Wave 5. People of colour households also showed higher odds of no hunger in some waves, notably Wave 3 (OR: 4.01; 95% CI: 1.69–9.55). However, NFNSS estimates for non-Black groups were imprecise and not statistically significant.

Socio-economic status (SES) emerged as a strong and consistent predictor of not experiencing hunger. Relative to the poorest households, the odds of not experiencing hunger increased monotonically across SES categories and over time. In Wave 1, households in the richest quintile had 3.75 times the odds of not experiencing hunger (95% CI: 2.43–5.79) compared with the poorest, rising to 9.45 (95% CI: 5.53–16.12) in Wave 2 and 10.06 (95% CI: 5.70–17.74) in Wave 5. A clear gradient was evident across all SES categories in most waves, with “Poorer,” “Middle,” “Richer,” and “Richest” households consistently showing higher odds of no hunger relative to the poorest. Importantly, this socio-economic gradient persisted in the NFNSS, where richer and richest households continued to have markedly higher odds of not experiencing hunger (e.g., richest: OR 6.80; 95% CI: 2.23–20.72). These findings highlight the persistence of income-related inequalities in food security during and after the pandemic.

Housing conditions were also associated with household food security. Compared with households living in formal houses or flats, those in informal/shack dwellings had lower odds of not experiencing hunger across most NIDS-CRAM waves, with ORs below 1, including 0.71 (95% CI: 0.50–1.00) in Wave 1 and 0.44 (95% CI: 0.28–0.69) in Wave 5. This indicates a heightened risk of hunger among households living in informal settlements, particularly by Wave 5. Households in traditional or mud structures also had lower odds of hunger, with a statistically significant association in Wave 5 (OR: 0.64; 95% CI: 0.44–0.93). NFNSS results for dwelling type were mixed and often imprecise, although the point estimate for informal dwellings (OR: 1.38; 95% CI: 0.57–3.31) suggested no clear post-pandemic difference relative to formal housing once other factors were controlled for.

Access to basic services showed less consistent associations with food security. Having electricity was not significantly associated with the odds of not experiencing hunger in most waves, with ORs close to 1 and confidence intervals spanning 1 across NIDS-CRAM and NFNSS. Access to piped water similarly showed limited and sometimes counterintuitive associations. While most waves showed no statistically significant effect, Wave 3 reported an OR of 0.45 (95% CI: 0.26–0.79), indicating lower odds of not experiencing hunger among households with piped water. Given the isolated nature of this finding and the absence of a consistent pattern across waves or in the NFNSS, this may reflect contextual factors or residual confounding rather than a stable relationship.

Education of the household head tended to be protective, although estimates were often imprecise. Compared with households where the head had no formal education, those with secondary education had higher odds of not experiencing hunger in Wave 1 (OR: 1.94; 95% CI: 1.05–3.57), and tertiary education was strongly associated with better food security in several waves, including Wave 1 (OR: 6.62; 95% CI: 1.40–31.33), Wave 2 (OR: 11.46; 95% CI: 2.19–59.91), and Wave 4 (OR: 5.46; 95% CI: 1.24–24.01). However, wide confidence intervals, particularly for tertiary education and NFNSS estimates, indicate small subsamples and suggest that these results should be interpreted cautiously.

Sex of the respondent and household size had more modest but relevant effects. Female respondents did not differ significantly from males in NIDS-CRAM Waves 1–5, with ORs close to 1. In the NFNSS, the odds of not experiencing hunger were higher among female respondents (OR: 1.93; 95% CI: 0.96–3.88), although this association was only marginally significant (*p* < 0.1). Household size was negatively associated with the odds of not experiencing hunger in Waves 4 and 5, with ORs of 0.89 (95% CI: 0.85–0.94) and 0.91 (95% CI: 0.87–0.95), respectively, indicating that larger households were more likely to experience hunger during the later stages of the pandemic. In contrast, NFNSS estimates suggested that larger households had slightly higher odds of not experiencing hunger (OR: 1.15; 95% CI: 1.02–1.30), suggesting possible shifts in household composition, coping strategies, or sample structure in the post-pandemic period. Respondent age showed small and inconsistent effects, with ORs close to 1 across all models and only a slight negative association with no hunger in Wave 2 (OR: 0.99; 95% CI: 0.98–1.00).

Overall, the regression results underscore the central role of socio-economic position, housing conditions, and household size in shaping the likelihood of not experiencing hunger during and after the COVID-19 pandemic. While some associations (e.g., employment status and basic services) were less stable over time, the persistent socio-economic gradient and the disadvantage associated with informal or traditional housing point to structural vulnerabilities that extend beyond the acute pandemic period.

### 3.3 Decomposition analysis

Table 5 shows that socio-economic inequality in not experiencing hunger persisted across all NIDS-CRAM waves, with positive Erreygers Concentration Indices (ranging from 0.15 to 0.25). These positive values indicate that households *not* experiencing hunger were disproportionately concentrated among wealthier socio-economic groups—reflecting pro-rich inequality in food security. The inequality was highest in Wave 5 (ECI = 0.2488) and lowest in the NFNSS (ECI = 0.1038), suggesting a moderate decline in pro-rich inequality in the post-pandemic period, though inequality remained substantial.

**Table 5:** Socio-economic inequality in households NOT experiencing hunger (No Hunger)—Erreygers concentration index for NIDS-CRAM waves 1–5 and NFNSS.

**Survey**	**Erreygers index**	**Robust Std. error**	***p*-value**	**Interpretation**
Wave 1	0.2029	0.0154	< 0.001	Pro-rich inequality (20.3%)
Wave 2	0.2035	0.0168	< 0.001	Pro-rich inequality (20.4%)
Wave 3	0.1463	0.0233	< 0.001	Pro-rich inequality (14.6%)
Wave 4	0.2098	0.0172	< 0.001	Pro-rich inequality (21.0%)
Wave 5	0.2488	0.0177	< 0.001	Pro-rich inequality (24.9%)
NFNSS	0.1038	0.0088	< 0.001	Pro-rich inequality (10.4%)

**Positive index values:** All indices are positive (ranging from 0.10 to 0.25), indicating **pro-rich inequality** in food security. Households NOT experiencing hunger are concentrated among wealthier socio-economic groups.

Table 6 presents the decomposition of these inequalities and highlights which factors contributed most to the observed patterns. Socio-economic status (SES) was by far the dominant contributor across all waves. In Wave 1, SES accounted for 78.94% of the inequality in food security, increasing to over 100% in Waves 2 and 4, and remaining high in Wave 5 (90.28%). Even in the NFNSS, SES explained 44.67% of all inequality. The persistent and large SES contribution confirms that wealth remained the primary determinant of unequal access to food security both during and after the pandemic.

**Table 6:** Erreygers decomposition analysis for households NOT experiencing hunger (No Hunger) for NIDS-CRAM waves 1–5 and NFNSS survey.

**Variable**	**Wave 1 (CI, %)**	**Wave 2 (CI, %)**	**Wave 3 (CI, %)**	**Wave 4 (CI, %)**	**Wave 5 (CI, %)**	**NFNSS (CI, %)**
Employment status	0.09, −0.31	0.09, −0.02	−0.00, 0.00	0.09, −0.53	0.10, 2.78	−0.04, 0.91
Race	0.12, 24.70	0.12, 25.98	0.13, 37.32	0.12, 16.21	0.14, 27.11	−0.03, 1.53
SES	0.40, 78.94	0.40, 103.22	0.33, 65.18	0.39, 100.12	0.38, 90.28	0.26, 44.67
Dwelling type	−0.23, 4.35	−0.22, 3.02	−0.27, 7.39	−0.24, 3.59	−0.23, 5.87	0.03, 0.43
Electricity access	−0.01, −0.91	−0.01, 0.92	−0.01, −2.32	−0.02, −0.06	−0.01, −1.37	0.00, 0.01
Piped water access	−0.04, 1.75	−0.03, 0.70	−0.04, 11.07	−0.04, 0.95	−0.04, −0.40	−0.01, −0.11
Respondent education	0.03, 5.88	0.03, 2.42	0.03, 2.48	0.03, 1.34	0.03, −1.13	−0.01, −0.21
Gender	−0.01, 0.16	−0.02, −0.11	−0.05, −0.15	−0.03, 0.51	−0.03, −0.43	0.01, 1.02
Household size	−0.02, 1.06	−0.02, 0.58	−0.07, 3.52	−0.00, 0.37	0.01, −0.56	0.04, 4.15
Respondent age	0.02, 2.46	0.02, −2.07	0.02, −1.09	0.02, −0.86	0.01, 0.37	0.01, −1.70
Unexplained variance	−18.09	−34.63	−23.40	−21.64	−22.50	9.29

CI represents the concentration index for each variable.

% represents the percentage contribution of each variable to inequality.

Race was the second major contributor to inequality during the NIDS-CRAM period. Race contributed between 24.70% (Wave 1) and 37.32% (Wave 3) to pro-rich inequality in no hunger, indicating that food-secure households were disproportionately concentrated among non-Black population groups. By Wave 5, the contribution had declined to 27.11%. In the NFNSS, however, the contribution of race was minimal (1.53%), with a negative concentration index (−0.03). This suggests that race became a much less salient dimension of inequality in the post-pandemic period compared with SES. Dwelling type showed consistent pro-poor inequality during the NIDS-CRAM waves, with negative concentration indices across Waves 1–5. This indicates that households in traditional and informal dwellings were less likely to report no hunger, accounting for 4%−7% of the inequality in food security across waves. In the NFNSS, the concentration index shifted to a small positive value (0.03), with a negligible contribution (0.43%), suggesting that dwelling type played a much smaller role in explaining inequality in the post-pandemic period.

Employment status contributed only modestly to inequality. In several waves, the percentage contribution hovered around zero and occasionally switched sign. For example, its contribution was −0.31% in Wave 1, −0.53% in Wave 4, and 2.78% in Wave 5. In the NFNSS, employment accounted for < 1% of total inequality, indicating a small and unstable role of labour-market status in shaping inequality in food security. Access to basic services (electricity and piped water) contributed very little to reducing inequality in no-hunger. Electricity access contributed between −0.91% and −2.32% in most waves, and only 0.01% in the NFNSS. Access to piped water showed a small pro-poor pattern in several waves, with contributions ranging from −0.40% to 11.07%. Overall, these service-related variables explained very little of the observed inequality.

Respondent education showed a small but consistently positive contribution to inequality during the pandemic, with contributions between 1%−6% across Waves 1–4. However, by Wave 5, the contribution was slightly negative (−1.13%), and NFNSS estimates were also small and negative (−0.21%). This suggests that education's role in shaping inequality in food security weakened over time. Gender and household size had a limited influence on inequality. Gender contributed less than ±1% in most waves, although the NFNSS showed a slight shift towards pro-rich inequality (1.02%). Household size contributed modestly, e.g., 3.52% in Wave 3, but remained small overall. In the NFNSS, household size accounted for 4.15%, suggesting a slight pro-rich pattern in post-pandemic food security.

The unexplained component of the decomposition, representing inequality not accounted for by observed factors, was sizeable and consistently negative in all NIDS-CRAM waves, ranging from −18.09% (Wave 1) to −34.63% (Wave 2). This indicates that a substantial share of the inequality during the pandemic was linked to unmeasured factors such as intra-household dynamics, informal safety nets, social capital, and unobserved economic shocks. In contrast, the NFNSS showed a positive unexplained contribution (9.29%), suggesting that in the post-pandemic period, a smaller proportion of inequality remained unexplained, possibly reflecting greater stability in the determinants of food security or improved measurement precision.

Overall, the decomposition analysis shows that socio-economic status remained the primary driver of pro-rich inequality in food security during and after the pandemic, while the importance of race and dwelling type diminished over time. Other demographic and service-related variables played only minor roles. These findings emphasise the enduring centrality of wealth-based inequalities in shaping household food security in South Africa.

### 3.4 Sensitivity analysis

A series of diagnostic and sensitivity checks were conducted to assess the robustness of the regression and inequality findings across all NIDS-CRAM waves and the NFNSS. Model specification tests showed no major functional-form misspecification, link tests indicated that the predicted values were significant while the squared predictions were not, except in Wave 1 and Wave 4, where mild specification concerns were detected but without substantive impact on the stability of coefficients (Supplementary Table 1). The Hosmer–Lemeshow goodness-of-fit test indicated adequate calibration in all models except Wave 4 (*p* = 0.037). However, the coefficients in Wave 4 remained consistent in direction and magnitude with those in adjacent waves. Discrimination statistics showed acceptable predictive performance across the NIDS-CRAM waves (AUC range: 0.6637–0.7180) and stronger performance for the NFNSS model (AUC = 0.7601), as illustrated in the ROC curves (Supplementary Figures 1–6).

Multicollinearity diagnostics also supported model stability (Supplementary Table 1). Mean VIF values ranged from 2.14 to 2.53 in NIDS-CRAM and were higher in the NFNSS model (5.10), primarily due to sparse educational categories rather than structural redundancy; significantly, no VIF exceeded conventional exclusion thresholds. Classification accuracy was high in all models, reflecting strong identification of households reporting no hunger, while low specificity in several waves was expected given the low prevalence of hunger. A key sensitivity check evaluated the stability of the socio-economic status (SES) gradient using alternative SES specifications (categorical vs. continuous) (Supplementary Table 2). The results remained consistent across all waves: higher SES was associated with increased odds of not experiencing hunger, and SES was jointly significant in every model (p < 0.001 in all waves except Wave 3: *p* = 0.016; NFNSS: *p* = 0.008). Differences in pseudo-*R*^2^ between categorical and continuous SES models were minimal (Δ < 0.01), confirming that the observed SES gradient is not sensitive to functional form.

Inequality estimates were similarly robust across sensitivity checks. The Erreygers Concentration Index remained positive in all waves (Table 5), indicating persistent pro-rich inequality in food security. Decomposition analysis (Table 6) confirmed that SES was consistently the dominant driver of inequality across all datasets and remained so under alternative SES specifications. Although the contributions of race, dwelling type, and household size varied in magnitude across survey waves, their direction and relative importance were stable. Additional sensitivity checks using the Wagstaff index yielded inequality patterns that closely mirrored Erreygers results, reinforcing the internal consistency of the inequality findings. Taken together, the diagnostic tests, SES specification checks, alternative inequality metrics, and decomposition results all demonstrate that the study's central conclusions are robust to model specification, weighting, SES operationalisation, and distributional assumptions.

## 4 Discussion

This study examined household hunger patterns before, during, and after the COVID-19 pandemic using five waves of NIDS-CRAM (2020–2021) and the post-pandemic (54). Unlike existing NIDS-CRAM studies that focus exclusively on the within-pandemic period (25–29), our study contributes new evidence by (i) extending analysis into the post-pandemic recovery phase, (ii) harmonising 7-day hunger measures across two independent national datasets, and (iii) applying the Erreygers Concentration Index and decomposition analysis to document how socio-economic inequalities evolved. This allowed us to compare both temporal trends and the structural determinants of hunger across distinct phases of the crisis.

Consistent with earlier analyses of NIDS-CRAM (25–29), our findings confirm that hunger peaked sharply during the first months of the lockdown, when 26.47% of households reported a hunger episode. This aligns with evidence that stringent movement restrictions disrupted incomes and food access, particularly among workers in the informal sector (36, 37). The decline in hunger observed from Waves 2–5 is consistent with studies such as Spaull et al. (19) and Alaba et al. (15), which show gradual stabilisation as economic activity resumed and emergency social protection measures expanded. However, the NFNSS data indicate a more substantial post-pandemic improvement (8.90% hunger), suggesting that recovery continued beyond the NIDS-CRAM period. Similar post-shock improvements have been documented in rural settings. For example, Chakona et al. (6) found that households in Mpumalanga increased their reliance on subsistence agriculture and natural resources as coping mechanisms, which helped stabilise food access despite job losses. Yet, our findings also highlight provinces where hunger remained elevated (the Northern Cape, North West andFree State), reflecting persistent structural vulnerabilities that limited recovery. Such heterogeneity is consistent with Ginsburg et al. (53), who showed that rural and return-migrant households experienced prolonged economic strain, while more mobile or urban-linked households recovered faster.

Employment status, household size, and SES remained central determinants of hunger, consistent with the South African literature (15, 40–44). The strong SES gradient, persistent across all five NIDS-CRAM waves and still evident in NFNSS reflects Sen's Entitlement Theory (45), whereby the loss of income erodes exchange entitlements and undermines the ability to command food. Our ECI results reinforce this, pro-rich inequality in food security intensified during the pandemic and declined only modestly thereafter. Larger households faced greater hunger risk during the pandemic (46, 47), reflecting higher consumption needs and reduced per-capita resources. However, NFNSS results suggest slightly improved food security among larger households in 2022, possibly reflecting shifts in household composition, shared grant income, or strengthened coping mechanisms. The changing vulnerability of female-headed households mirrors wider evidence. During early waves, women benefited from grant top-ups (48), but post-pandemic NFNSS patterns reflect heightened labour market vulnerability among women (49, 50), consistent with Nwosu and Oyenubi (8). This underscores the gendered nature of economic shocks and highlights the need for gender-responsive recovery policies.

Racial disparities in food security were pronounced during the early pandemic waves, consistent with enduring apartheid-era inequalities (18, 51, 52). However, NFNSS results show that race contributed minimally to inequality in 2022, suggesting partial narrowing of racial food-security gaps. This finding aligns with emerging post-pandemic evidence showing that socio-economic rather than racial factors have become more salient in determining recovery trajectories. Housing and service access remained persistent structural vulnerabilities. Households in informal and traditional dwellings consistently faced a greater risk of hunger during the pandemic, supporting the social determinants framework that links poor housing conditions to constrained coping capacity and reduced resilience.

Across all NIDS-CRAM waves, ECI values were positive (0.15–0.25), showing persistent pro-rich inequality. SES was the dominant contributor to inequality (45%−100%), consistent with patterns described by Alaba et al. (15). Race and dwelling type played secondary roles and diminished in importance post-pandemic. The large negative unexplained component in NIDS-CRAM waves reflects unmeasured pandemic shocks (food price volatility, informal transfers, community safety nets), while the smaller unexplained share in NFNSS indicates greater stability in the determinants of food security by 2022.

The provincial contrasts in our findings align with emerging evidence from rural areas. Rusere et al. (38) show that rural households in Mpumalanga leveraged natural resources and subsistence agriculture to buffer shocks, which explains why some regions recovered faster. Conversely, Ginsburg et al. (53) documented increased vulnerability among return migrants and rural households with limited livelihood diversification, consistent with our findings for the Northern Cape and North West. These parallels reinforce that pandemic impacts were not uniform, and that recovery depended on livelihood diversification, access to social grants, migration patterns, and ecological resource availability (39).

### 4.1 Strengthens and limitations

#### 4.1.1 Strengths of the Study

This study has several strengths that enhance its contribution to the evidence base on food security in South Africa during and after the COVID-19 pandemic. First, it is one of the few analyses to cover both the pandemic period and the post-pandemic recovery phase using two large, nationally representative datasets, NIDS-CRAM and the NFNSS. By harmonising a comparable 7-day household hunger indicator across these independent surveys and recoding it as a “no hunger” outcome, the study provides a coherent and policy-relevant picture of how household food security evolved beyond the initial crisis period. Second, the longitudinal structure of NIDS-CRAM, with five waves spanning different stages of lockdown and reopening, allows for tracking changes in hunger and inequality over time that single-wave or purely cross-sectional studies cannot. The inclusion of NFNSS extends this trajectory into 2022, offering a post-pandemic snapshot against which the pandemic-period dynamics can be interpreted.

Third, the analytical strategy goes beyond simple descriptive comparisons. We estimated theory-driven multivariable logistic regression models for each NIDS-CRAM wave and for NFNSS, using a consistent set of covariates grounded in existing frameworks on poverty, vulnerability, and food access. To address concerns about model validity, we implemented a comprehensive diagnostic suite: link tests for model specification, Hosmer–Lemeshow goodness-of-fit tests on unweighted models, ROC curves to assess discrimination, and multicollinearity checks using variance inflation factors. We also tested the joint significance of SES categories and conducted sensitivity analyses modelling SES as an ordinal variable. These steps strengthen confidence that the observed associations are not artefacts of model mis-specification.

Fourth, the study applies the Erreygers Concentration Index and decomposition to quantify socio-economic inequality in the “no hunger” outcome. We also computed Wagstaff indices as a sensitivity check and used clustered standard errors to account for survey design. This dual approach to measuring inequality, combined with decomposition, provides a richer understanding of how different determinants, particularly socio-economic status, contribute to unequal distributions of food security over time. Fifth, the analysis incorporates provincial estimates using appropriate survey weights for both NIDS-CRAM and NFNSS, enabling a geographically disaggregated view of recovery that can be linked to emerging evidence from rural and peri-urban settings. Finally, the study's explicit focus on structural vulnerability housing type, access to electricity and piped water, race, gender, and household size ensures that the findings are situated within South Africa's long-standing patterns of inequality, enhancing both their interpretability and their policy relevance.

#### 4.1.2 Limitations

Our findings must be interpreted while taking several limitations into account. First, NIDS-CRAM and NFNSS differ in sampling frames, data collection modes, and question framing, harmonisation allows descriptive comparison but not strict equivalence. Second, hunger was operationalised as a binary 7-day measure, which does not capture severity or the broader dimensions of food insecurity. Third, some NFNSS provincial hunger estimates may reflect variability in weighting, underlying sample differences, or contextual factors affecting measurement. Fourth, logistic regression results may be affected by omitted variables such as food prices, income shocks, and access to grants. Finally, the observational nature of the data limits causal inference.

## 5 Conclusion

This study used five waves of NIDS-CRAM (2020–2021) and the post-pandemic Simelane et al. (54) to track household hunger in South Africa, showing a sharp rise in hunger during the early lockdown followed by partial recovery, with the share of households reporting no hunger increasing over time but remaining uneven across provinces and groups. Socio-economic status was the strongest and most persistent determinant of being hunger-free, and the Erreygers Concentration Index and decomposition analyses revealed enduring pro-rich inequality in food security, even as the relative contributions of race and dwelling type declined over time. While employment, household size, gender, and housing conditions also shaped vulnerability, their effects were less consistent, and some provinces, particularly the Northern Cape, Free State, and North West, remained comparatively food insecure in 2022. Taken together, these findings suggest that although the food security shock linked to COVID-19 has eased, deep-rooted socio-economic inequalities continue to structure who can avoid hunger, underscoring the need for sustained, structural policy measures that strengthen social protection, support low-income and informal workers, and address spatial and infrastructural deficits to promote more equitable and resilient food security.

## Data Availability

Publicly available datasets were analyzed in this study. This data can be found here: The NIDS-CRAM dataset used in this study is publicly available online at https://www.nids.uct.ac.za/nids-cram. The NFNSS dataset is not publicly available due to data-sharing restrictions but can be accessed upon reasonable request through Professor Charles Hongoro.

## Footnotes

1 NIDS ran between 2008 and 2017 with a nationally representative sample of over 28,000 individuals in 7,300 households over time and reported on their livelihoods and wellbeing across South Africa. The latest round of in-person data collection (Wave 5) was conducted in 2017 (SALDRU, 2018). The follow-up telephonic survey (NIDS-CRAM) was available in 10 official languages.
