# Supplementary material for: Impact of COVID-19 on household hunger and socio-economic inequality in South Africa: a comparative analysis using NIDS-CRAM (2020–2021) and NFNSS 2022 data
Source: Front Public Health. 2026 Jan 12;13:1736131. doi: 10.3389/fpubh.2025.1736131 (PMC12832473; doi:10.3389/fpubh.2025.1736131)
Supplement: Supplementary file 1 [file Table_1.docx]

Supplementary Table 1: Model Fit Diagnostics Across Survey Waves

| Diagnostic Test | Wave 1 | Wave 2 | Wave 3 | Wave 4 | Wave 5 | NFNSS |
| --- | --- | --- | --- | --- | --- | --- |
| **Model Fit Statistics** |  |  |  |  |  |  |
| Wald χ² (df) | 135.96 (19) | 165.65 (19) | 74.40 (16) | 182.65 (19) | 237.03 (19) | 60.85 (17) |
| Wald χ² p-value | <0.001 | <0.001 | <0.001 | <0.001 | <0.001 | <0.001 |
| Pseudo R² | 0.0856 | 0.1242 | 0.1344 | 0.1256 | 0.1482 | 0.1139 |
| Log Pseudolikelihood | -10,645,409 | -8,797,982.6 | -2,761,225 | -10,475,777 | -10,303,126 | -463.485 |
| **Specification Tests** |  |  |  |  |  |  |
| Link Test: _hat p-value | 0.018 | <0.001 | 0.030 | 0.002 | <0.001 | 0.902 |
| Link Test: _hatsq p-value | 0.571 | 0.954 | 0.873 | 0.141 | 0.339 | 0.288 |
| Hosmer-Lemeshow χ² (8 df) | 7.29 | 3.36 | 11.14 | 16.40 | 6.51 | 8.25 |
| Hosmer-Lemeshow p-value | 0.505 | 0.910 | 0.194 | 0.037 | 0.590 | 0.410 |
| **Discrimination** |  |  |  |  |  |  |
| Area Under ROC Curve (AUC) | 0.6637 | 0.6988 | 0.6881 | 0.7058 | 0.7180 | 0.7601 |
| **Classification Accuracy** |  |  |  |  |  |  |
| Overall Correct (%) | 99.11 | 99.34 | 99.12 | 99.06 | 97.68 | 100.00 |
| Sensitivity (%)* | 99.34 | 99.71 | 99.28 | 99.36 | 98.78 | 100.00 |
| Specificity (%)* | 1.64 | 2.38 | 1.94 | 97.71 | 7.38 | 100.00 |
| **Multicollinearity** |  |  |  |  |  |  |
| Mean Variance Inflation Factor (VIF) | 2.14 | 2.15 | 2.53 | 2.17 | 2.16 | 5.10 |
| Maximum VIF | 8.86 (Educ) | 8.76 (Educ) | 11.89 (Educ) | 8.54 (Educ) | 8.58 (Educ) | 33.32 (Educ) |
| **SES Joint Significance** |  |  |  |  |  |  |
| Wald χ² (4 df) for SES | 42.22 | 70.07 | 12.26 | 77.81 | 89.43 | 13.70 |
| p-value for SES | <0.001 | <0.001 | 0.016 | <0.001 | <0.001 | 0.008 |

*Note: Classification based on 0.5 probability cutoff. Sensitivity = correctly classified households with no hunger; Specificity = correctly classified households with hunger.*

**Supplementary Table 2: Sensitivity of Results to Socioeconomic Status (SES) Specification**

| Model Specification | Wave 1 | Wave 2 | Wave 3 | Wave 4 | Wave 5 | NFNSS |
| --- | --- | --- | --- | --- | --- | --- |
| **A. Categorical SES (Ref: Poorest Quintile)** |  |  |  |  |  |  |
| Odds Ratio (95% CI) |  |  |  |  |  |  |
| Poorer (Q2) | 1.06 (0.77-1.47) | 1.58 (1.09-2.31) | 1.07 (0.58-2.00) | 2.69 (1.89-3.83) | 1.55 (1.07-2.23) | 2.98 (1.18-7.53) |
| Middle (Q3) | 1.62 (1.14-2.29) | 2.00 (1.31-3.05) | 1.29 (0.68-2.44) | 2.19 (1.41-3.41) | 2.02 (1.37-2.99) | 3.46 (1.22-9.80) |
| Richer (Q4) | 1.51 (1.07-2.13) | 2.67 (1.73-4.11) | 1.86 (0.88-3.90) | 3.05 (2.02-4.62) | 4.96 (3.22-7.64) | 2.90 (1.13-7.42) |
| Richest (Q5) | 3.75 (2.43-5.79) | 9.45 (5.53-16.12) | 5.29 (1.91-14.60) | 11.93 (6.62-21.48) | 10.06 (5.70-17.74) | 6.80 (2.23-20.72) |
| Joint test p-value | <0.001 | <0.001 | 0.016 | <0.001 | <0.001 | 0.008 |
| **B. Continuous SES (Per Quintile Increase)** |  |  |  |  |  |  |
| Odds Ratio (95% CI) | 1.30 (1.19-1.42) | 1.54 (1.38-1.72) | 1.35 (1.12-1.62) |  | 1.71 (1.53-1.90) | 1.50 (1.16-1.95) |
| Wald test p-value | <0.001 | <0.001 | 0.001 |  | <0.001 | 0.002 |
| **C. Model Fit Comparison** |  |  |  |  |  |  |
| Pseudo R² (Categorical) | 0.0856 | 0.1242 | 0.1344 | 0.1256 | 0.1482 | 0.1139 |
| Pseudo R² (Continuous) | 0.0803 | 0.1190 | 0.1283 |  | 0.1448 | 0.1047 |
| Δ Pseudo R² | -0.0053 | -0.0052 | -0.0061 |  | -0.0034 | -0.0092 |

Note: *All models include full covariate set: employment status, race, dwelling type, electricity access, piped water access, education, gender, household size, and respondent age.* *Odds ratios >1 indicate higher odds of household food security (no hunger).*

Supplementary figures 1-6: Receiver Operating Characteristic (ROC) curves for logistic regression models predicting household food security (no household hunger) across NIDS-CRAM Waves 1–5 and the NFNSS.

| **Wave 1** | **Wave 2** | **Wave 3** |
| --- | --- | --- |
| 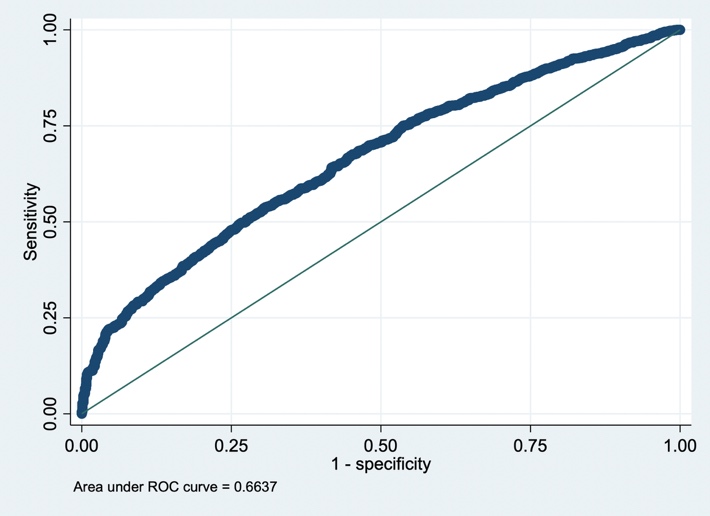 | 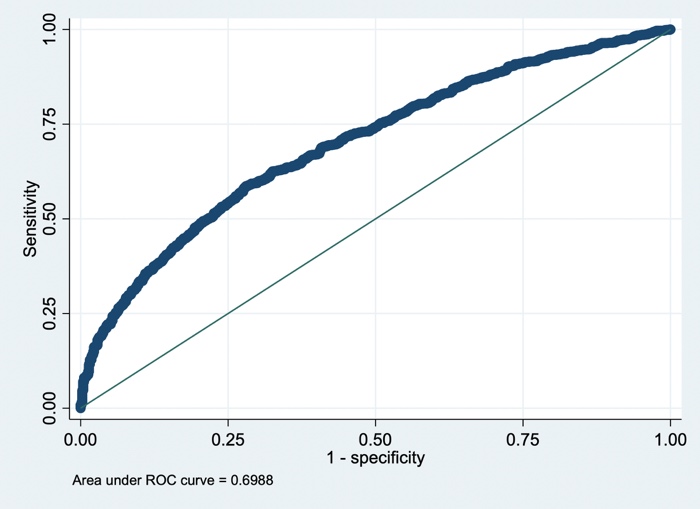 | 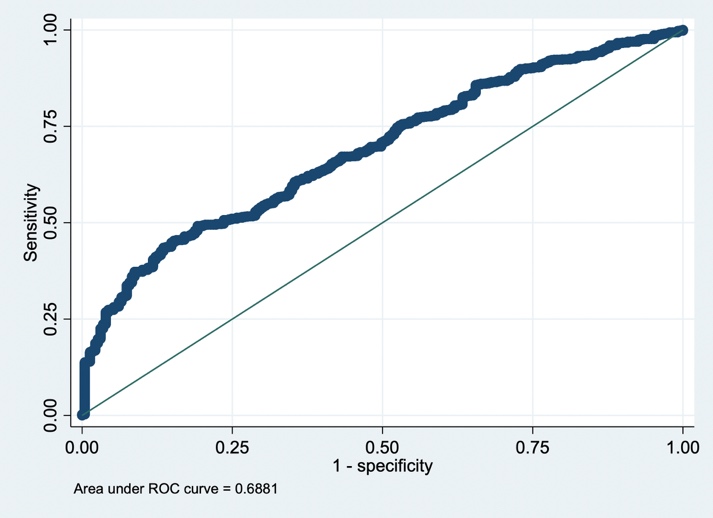 |
| **Wave 4** | **Wave 5** | **NFNSS** |
| 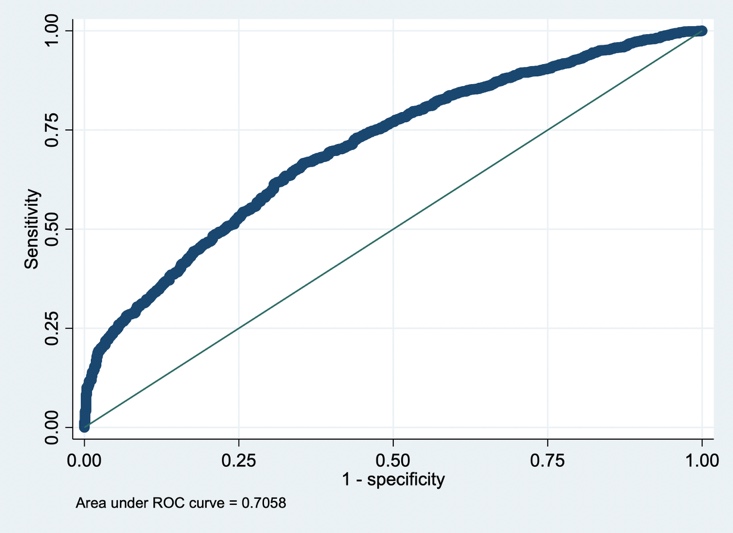 | 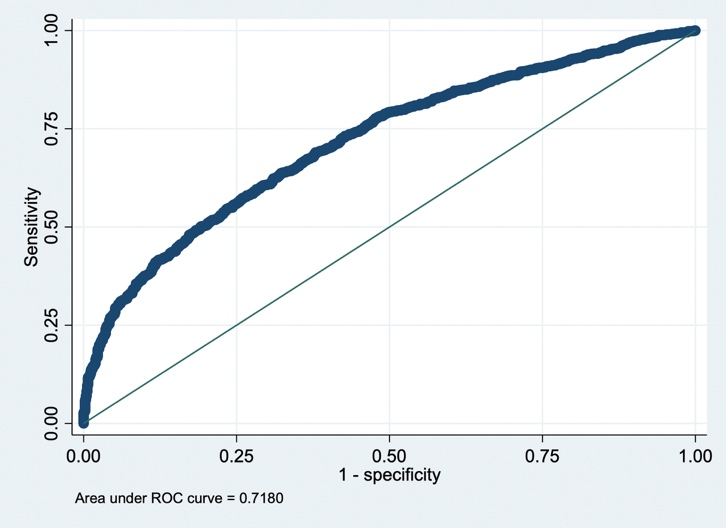 | 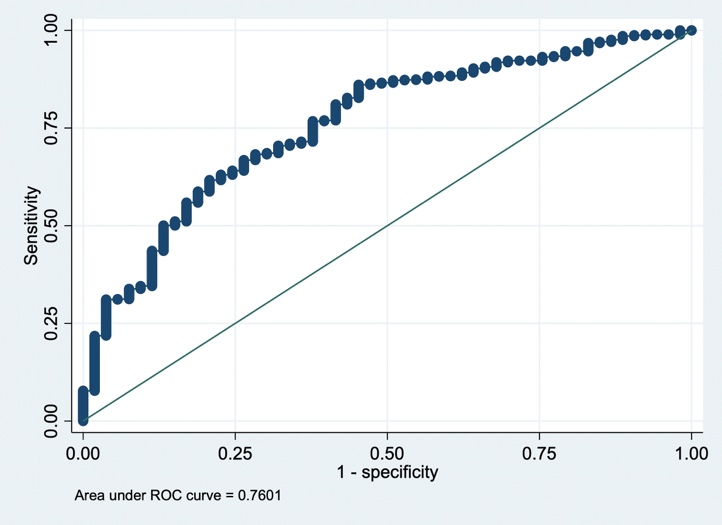 |
